# Cerebrospinal Fluid Neurogranin Levels and Cognitive Function in Alzheimer’s Disease and Frontotemporal Lobar Degeneration – Data from CBAS

**DOI:** 10.1002/alz.093271

**Published:** 2025-01-09

**Authors:** Vanesa Jurasova, Ross Andel, Alzbeta Katonova, Terezie Zuntychová, Hana Horakova, Martin Vyhnalek, Vaclav Matoska, Katerina Veverova, Jakub Hort

**Affiliations:** ^1^ Memory Clinic, Department of Neurology, 2nd Faculty of Medicine, Charles University and Motol University Hospital, Prague Czech Republic; ^2^ Edson College of Nursing and Health Innovation, Arizona State University, Phoenix, AZ USA; ^3^ International Clinical Research Center, St. Anne’s University Hospital, Brno Czech Republic; ^4^ Department of Clinical Biochemistry, Hematology and Immunology, Homolka Hospital, Prague Czech Republic

## Abstract

**Background:**

Synaptic dysfunction occurs in the early stages of neurodegenerative diseases and leads to the breakdown of connections within neuronal networks. Therefore, biomarkers reflecting this process, such as neurogranin (Ng), might be useful to study disease pathophysiology and aid in diagnostics. Ng is crucial for long‐term potentiation and memory consolidation, and plays a central role in synaptic plasticity. It has been reported that Ng levels in cerebrospinal fluid (CSF) are elevated in Alzheimer’s disease (AD), but also in frontotemporal lobar degeneration (FTLD). The aim of this study was to investigate levels of Ng in AD and FTLD, and to evaluate Ng levels and cognitive function in AD.

**Method:**

Participants with aMCI due to AD (n = 109), AD dementia (n = 67), MCI due to FTLD (n = 25), and FTLD dementia (n = 29) were recruited from the Czech Brain Aging Study. All individuals underwent clinical evaluation, MRI and analysis of biomarkers in CSF (Aß42, pTau181, tTau). Levels of Ng in CSF were measured using the LUMIPULSE® G600II instrument (Fujirebio, Ghent, Belgium). A one‐way analysis of covariance (ANCOVA) with post hoc Tukey’s HSD and multivariate linear regression with age, sex, and years of education as covariates were performed using the R statistical software.

**Result:**

Patients with aMCI due to AD and AD dementia had significantly higher Ng levels compared to patients with MCI due to FTLD and FTLD dementia. The association between Ng levels and assessments in memory, attention, executive, language, and visuospatial domains did not statistically differ between patients with AD dementia and those with aMCI due to AD.

**Conclusion:**

Ng levels are higher in AD compared to FTLD both in aMCI and dementia stage, but are not related to cognition in AD individuals. Our results indicate that higher levels of Ng are specifically associated with AD pathology and these changes are more prominent in aMCI due to AD suggesting Ng as an early biomarker of AD.

